# A framework for the computational prediction and analysis of non-coding RNAs in microbial environmental populations and their experimental validation

**DOI:** 10.1038/s41396-020-0658-7

**Published:** 2020-04-28

**Authors:** Steffen C. Lott, Karsten Voigt, S. Joke Lambrecht, Wolfgang R. Hess, Claudia Steglich

**Affiliations:** University of Freiburg, Faculty of Biology, D-79104 Freiburg, Germany

**Keywords:** Non-coding RNAs, Water microbiology

## Abstract

Small regulatory RNAs and antisense RNAs play important roles in the regulation of gene expression in bacteria but are underexplored, especially in natural populations. While environmentally relevant microbes often are not amenable to genetic manipulation or cannot be cultivated in the laboratory, extensive metagenomic and metatranscriptomic datasets for these organisms might be available. Hence, dedicated workflows for specific analyses are needed to fully benefit from this information. Here, we identified abundant sRNAs from oceanic environmental populations of the ecologically important primary producer *Prochlorococcus* starting from a metatranscriptomic differential RNA-Seq (mdRNA-Seq) dataset. We tracked their homologs in laboratory isolates, and we provide a framework for their further detailed characterization. Several of the experimentally validated sRNAs responded to ecologically relevant changes in cultivation conditions. The expression of the here newly discovered sRNA Yfr28 was highly stimulated in low-nitrogen conditions. Its predicted top targets include mRNAs encoding cell division proteins, a sigma factor, and several enzymes and transporters, suggesting a pivotal role of Yfr28 in the coordination of primary metabolism and cell division. A *cis*-encoded antisense RNA was identified as a possible positive regulator of *atpF* encoding subunit b’ of the ATP synthase complex. The presented workflow will also be useful for other environmentally relevant microorganisms for which experimental validation abilities are frequently limiting although there is wealth of sequence information available.

## Introduction

Non-coding (nc)RNAs such as *trans*-acting small (s)RNAs and antisense RNAs (asRNAs) that overlap with other transcripts in *cis* are important components in the global control of gene expression in bacteria. Their functional roles have been studied in detail in genetically tractable bacteria such as *Escherichia coli*, *Bacillus subtilis*, *Staphylococcus aureus*, *Pseudomonas aeruginosa,* and *Vibrio cholera* (for reviews see [1–5]). However, there are many other important groups of bacteria for which no genetic tools exist or which even cannot be cultivated in the laboratory, enormously hampering the exploration of their respective sets of regulatory RNAs. On the other hand, especially for environmentally relevant microorganisms, frequently vast quantities of sequence data are available from extensive metagenomic and metatranscriptomic surveys, and such information can be expected to become even more readily available in the future.

The marine cyanobacterium *Prochlorococcus* is the most abundant phototroph throughout the euphotic zone of the vast oligotrophic areas of the oceans between 40°N and 40°S [6]. On the basis of extrapolations, it has been calculated that there are a total of 10^27^ *Prochlorococcus* cells on Earth, which fix an estimated four gigatons of carbon per year, comparable to the net primary production of all croplands in the world [7]. Phylogenetically distinct ecotypes of *Prochlorococcus* inhabit the oceans [8], which can be divided into two groups according to their adaptation to low light (LL) or high light (HL) conditions [9]. At the northern tip of the Gulf of Aqaba (Station A, 29°28′N 34°55′E) - the sampling site of this study – *Prochlorococcus* cells can reach a density of up to 2 × 10^5^ per ml during the summer at the height of stratification [10] and are dominated by HL clade II (eMIT9312) and LL clade noncultured 1 (NC1), which is most closely related to LL I (eNATL2a) [11].

*Prochlorococcus* genome sizes vary between 1.62 and 2.68 Mbp [12], which is very small for a free-living photoautotroph. The highly streamlined genomes of *Prochlorococcus* have been interpreted as an adaptation to ultralow nutrient conditions [13]. The reduced genome size correlates with a small number of protein regulators [8]. Therefore, the control of gene expression by sRNA regulators might have been particularly important during the evolution of this phylum. Indeed, a relatively large number of sRNAs have been found in *Prochlorococcus* by computational prediction, microarray analysis and high-throughput sequencing [14–16]. However, despite the wealth of available genome information, the knowledge of the transcriptional architecture and the numbers and types of potential regulatory RNA molecules is still largely fragmentary and limited to the HL-adapted MED4 strain [15] and the LL-adapted MIT9313 strain [16].

We therefore collected seawater samples from three different depths in the Gulf of Aqaba, Red Sea, and extracted RNA for RNA-seq. The sampling location was chosen because it is (i) well characterized, (ii) has been monitored for decades (including physicochemical properties and the planktonic community composition) and (iii) is well known for the high abundance of *Prochlorococcus* during summer, allowing the straightforward collection of sufficient quantities of cells.

The identification of key RNA regulators within microbial communities is still challenging, and a convenient pipeline for *in silico* analysis and experimental verification has not yet been deployed. Here, we present a computational workflow for the identification of putative sRNAs based on the analysis of environmental transcriptome datasets and their experimental validation. These analyses led to the discovery of several new *Prochlorococcus*-specific sRNAs, which are likely of ecological importance.

## Materials and methods

### Preprocessing and global read assignment

For the identification of new sRNAs, we used three datasets from samples that were collected at station A in the Red Sea at depths of 60, 100, and 130 m [17]. The sequencing and preprocessing of the reads included quality control, quality trimming, and removal of ribosomal RNA reads, as described previously [17]. The preprocessed datasets were aligned to the NCBI nt database using discontiguous MegaBLAST [18].

We utilized MEGAN’s built-in “LCA” (Lowest Common Ancestor) function to achieve a higher taxonomic assignment resolution by reassigning multimapped reads to a higher taxonomic level [19]. Analyses were performed with the default settings. For normalization and selection of taxa of interest, we extracted global taxonomic tree information (number of assigned reads per taxon) from MEGAN [20]. Based on the three datasets, we computed a comparative weighted Venn tree for the Cyanobacteria phylum focusing on *Prochlorococcus* with CoVennTree v1.6.0 [20].

### Computational identification of sRNA candidates

From the MEGAN output, we extracted *Prochlorococcus*-related reads in multi-FASTA format. For the computational analysis of sRNA candidates, we merged the three multi-FASTA datasets (60, 100, and 130 m) into a single file, which was further condensed into contigs using the de novo assembler Trinity v2.1.1 [21]. Next, the contigs were mapped against the well-annotated genomes of two *Prochlorococcus* model strains, MED4 (accession number: BX548174) and NATL2A (accession number: CP000095), using segemehl [22, 23]. Only contigs that did not overlap at all with a protein-coding gene were kept, and these contigs were used as the input to search for homologs throughout the entire cyanobacterial phylum using the GLASSgo v1.220 algorithm [24]. The putative sRNA homologs predicted by GLASSgo were aligned with Clustal Omega v1.2.3 and scored with RNAz v2.1, which computes a *z*-score based on secondary structure conservation and thermodynamic stability [25, 26], using the default settings for both the Clustal Omega and RNAz. Trinity results (number of reads per contig), and the GLASSgo outputs in conjunction with the RNAz predictions were compiled into master tables (Tables S2, S3 for NATL2A and MED4, respectively).

### Culturing, RNA preparation and northern blot analysis

*Prochlorococcus* NATL2A and MED4 cultures were grown at 22 °C in AMP1 medium [27] under 10–30 µmol quanta m^−2^ s^−1^ of continuous white cool light to cell densities of 1–3 × 10^8^ cells per ml. Stress experiments were performed as described previously [16]. In brief, the cells were subjected to several stress conditions for 30 min: light stress (light shifts from 10 µE to 100 µE (NATL2A) or 30 µE to 300 µE (MED4) or darkness) and temperature stress (shifts from 22 °C to 12 °C or 32 °C, respectively). For nitrogen starvation, the cells were washed twice in nitrogen-free medium and grown in minus N medium for 2 days. Iron depletion was induced via the addition of 0.6 µM DFB 2 days before sampling. The cells were harvested via filtration onto Supor-450 membranes, snap frozen in liquid nitrogen in tubes containing 2 ml of PGTX buffer [28] and subsequently stored at – 80 °C. Total RNA was extracted following the hot phenol method [29]. Northern hybridizations were performed as described previously [30] using 5 µg (small polyacrylamide gels and denaturing agarose gels), 10 µg (large polyacrylamide gels) or 50 µg (identification of new sRNAs on large polyacrylamide gels) of total RNA per sample. The primers used for the generation of specific probes are listed in Table S1.

### Primer extension

Transcript templates for in vitro RNA synthesis were generated from purified PCR products or annealed complementary oligonucleotides using primers #17–20 (Table S1). The desired RNAs were transcribed using a MEGAshortscript Kit (ThermoFisher Scientific), and residual DNA was removed by TURBO DNase I treatment, with both steps performed according to the manufacturer’s instructions. RNA was purified using RNA Clean & Concentrator columns (Zymo Research) following the manufacturer’s instructions. If required, in vitro-transcribed RNA was separated on 7 M urea-10% polyacrylamide gels or on 2% nondenaturing agarose gels, and full-length fragments were excised and purified using either a ZR small-RNA PAGE Recovery Kit (Zymo Research) or a NucleoSpin Gel and PCR-Clean-up kit (Macherey-Nagel) according to the manufacturer’s instructions. The ppc_RT primer (#21, Table S1) was labeled as described previously [30]. Annealing mixtures containing 0.2 pmol of in vitro-synthesized *ppc* target RNA and 2 pmol of the 5′ end-labeled primer #21 (Table S1) without or with 40/80/160 pmol of in vitro-synthesized sRNA Yfr28 or 160 pmol of in vitro-synthesized sRNA Yfr2 were heated for 10 min at 70 °C and then chilled on ice for at least 5 min. cDNA synthesis was performed for 2 h at 30 °C using SuperScript III Reverse Transcriptase (ThermoFisher Scientific) according to the manufacturer’s instructions. The reaction was inactivated by incubation for 15 min at 70 °C, followed by RNase H treatment for 20 min at 37 °C and a final heat inactivation step of 5 min at 95 °C in RNA loading buffer. DNA sequencing ladder reactions were performed with the same 5′ end-labeled primer used for cDNA synthesis and the same template DNA employed for the in vitro synthesis of the target RNA using a USB Thermo Sequenase Cycle Sequencing Kit (Affymetrix). The primer extension products and sequencing reactions were separated on 8.3 M urea-6% polyacrylamide sequencing gels, and the vacuum-dried gels were exposed to imaging plates. Signals were visualized using a Typhoon FLA 9500 instrument (GE Healthcare) with Quantity One software (Bio-Rad).

## Results and discussion

### Workflow for the identification and characterization of sRNAs in environmentally relevant bacteria

**Fig. 1:**
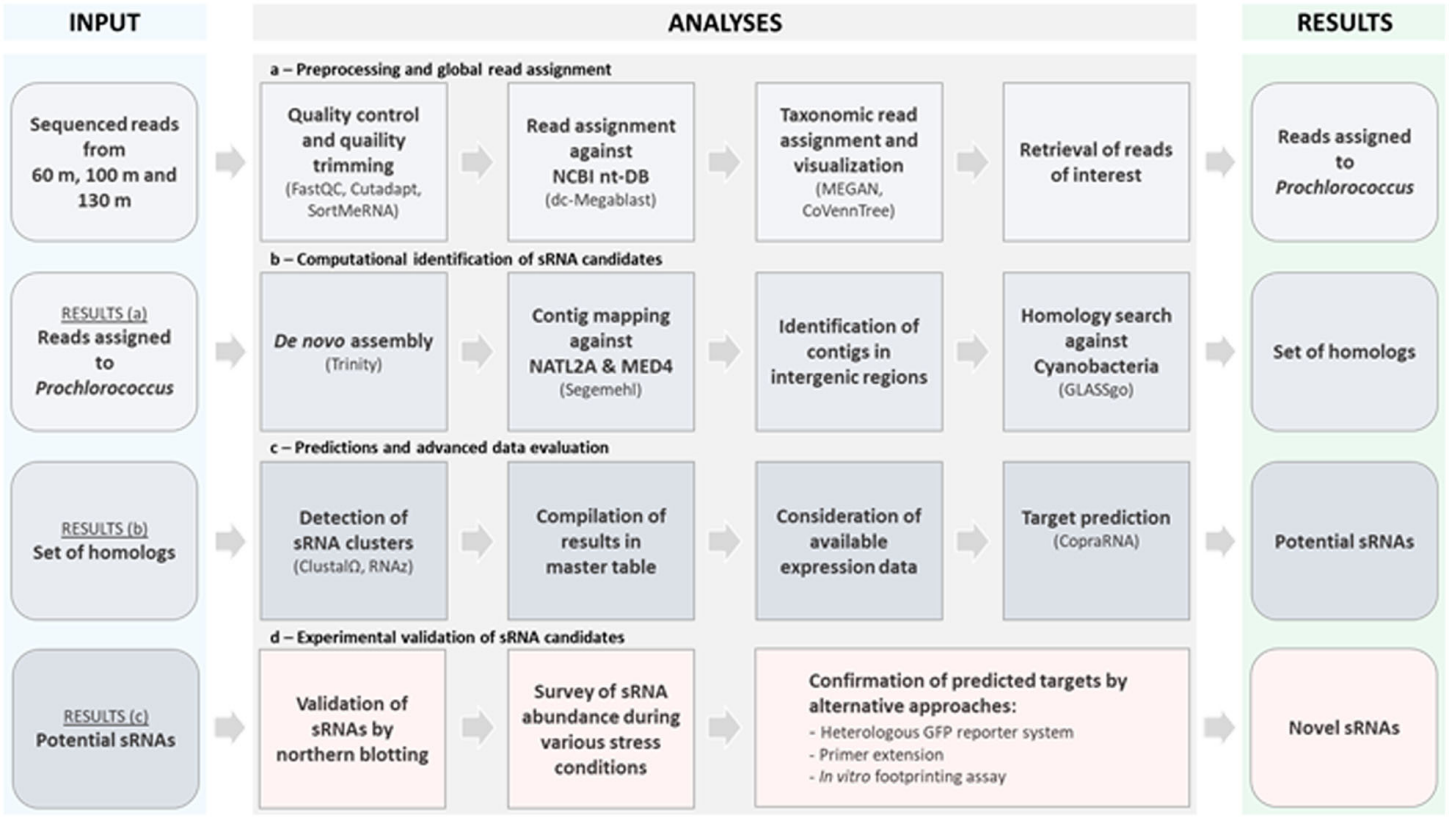
Workflow for the identification of sRNA candidates through mdRNA-Seq analysis of *Prochlorococcus*-related cDNA sequences from environmental samples. The workflow was modified from previous publications [32, 54] and follows the recommendations given in the reference [55].

**Fig. 2:**
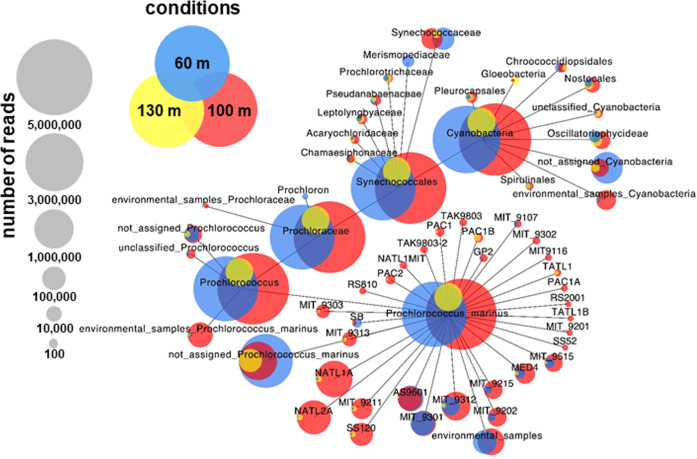
Metatranscriptomic read coverage from the mdRNA-Seq analysis of *Prochlorococcus* genome sequences from samples from three different depths in the Red Sea. Samples were collected from 60 m (blue circles), 100 m (red circles), and 130 m (yellow circles) water depths. After the initial bioinformatic analysis (see workflow in Fig. 1), the weighted Venn tree was computed using CoVennTree [20]. The numbers in parentheses refer to weighted Venn decomposition values. To enable comparisons, each library was normalized to 10,000,000 reads. Of the entire computed CoVennTree graph only the cyanobacterial phylum is shown.

Environmental samples were collected at Station A in the northern Gulf of Aqaba, Red Sea [17]. During the preparation of these samples, the RNA was treated with terminator exonuclease prior to library preparation [17] according to the differential RNA-Seq protocol [31]. This modification has several advantages, as it reduces the number of ribosomal RNAs and other processed transcripts, allows the precise mapping of transcriptional start sites, yields specific information about sRNAs and has been demonstrated to work well on environmental samples [32]. Here, all the sequenced reads from the samples obtained from the three different depths (60, 100, and 130 m) were processed as summarized in the workflow in Fig. 1. After taxonomic read assignment, we focused on the reads that could be assigned to genomic sequences belonging to the genus *Prochlorococcus*. These constituted 14.6, 8.1, and 0.7% of all reads from 60, 100, and 130 m. The known classification of *Prochlorococcus* according to ecotype was clearly visible in the mapping of the metatranscriptomic reads to distinct genomic sequences. Whereas the majority of *Prochlorococcus*-specific reads from 60 m mapped to *Prochlorococcus* of the HL ecotype, such as the AS9601, MIT9301, MIT9312, MIT9202, MIT9215, MED4, and MIT9515 strains, most of the reads from the two greater depths mapped to representatives of the LL ecotypes, such as the NATL1A, NATL2A, MIT9211, and SS120 strains (Fig. 2). Similar distribution profiles were detected by Shibl et al. [11] based on 16S–23 S rRNA internal transcribed spacer clone libraries that were generated from samples collected in September and October 2011 throughout the water column at the northern and southern ends of the Red Sea.

The next steps of the analysis included reassignment of the *Prochlorococcus*-related reads against the well-annotated genome sequences of two *Prochlorococcus* model strains, NATL2A (Files S1 and S2) [33] and MED4 [34]. We choose the LL strain NATL2A because this was one of the strains to which most of the reads could be assigned to within the LL clade, and the HL strain MED4 because this is the strain for which most information on *Prochlorococcus* sRNAs is available. The reads that mapped to intergenic regions (IGRs) and to ncRNAs (including housekeeping genes such as tRNAs, *rnpB, ffs*, and *yfr*s) were collected, unified and used as the input to search for homologs throughout the cyanobacterial phylum using the GLASSgo algorithm [24], which yielded 389 and 982 IGRs and annotated ncRNAs for NATL2A and MED4 (Tables S2 and S3). GLASSgo homologs were subjected to RNAz, which scores multiple sequence alignments of sRNA candidates based on secondary structure conservation and thermodynamic stability [25]. Based on the *z*-score (≤ −1), a total of 104 and 152 IGRs in NATL2A and MED4 were considered potential sRNA candidates (Tables S2, S3). Among these candidates, 34 in NATL2A and 42 in MED4 were previously annotated as tRNAs, housekeeping RNAs such as tmRNA and *ffs*, *yfr*s or ribosomal RNAs (Tables S2, S3). Many of the RNA classes were also found when searching in the RFAM database [35, 36] (Tables S2, S3). In total, we detected 24 of the 30 previously identified *Prochlorococcus* sRNAs [14–16] in the three datasets (Table S5) suggesting that our workflow is very suitable for the discovery of ncRNAs. The phylogenetic distribution of the MED4 and NATL2A sRNA candidates in other *Prochlorococcus* clades, cyanobacteria and other bacteria is given in Table S4.

Following manual inspection, selected sRNA candidates were experimentally validated and characterized in more detail, and sets of homologous sRNAs were subjected to target prediction in parallel (Tables S6, S7) using the CopraRNA package [37, 38]. The results will be presented in the following section.

### Validation and characterization of predicted sRNAs and asRNAs in laboratory isolates

**Fig. 3:**
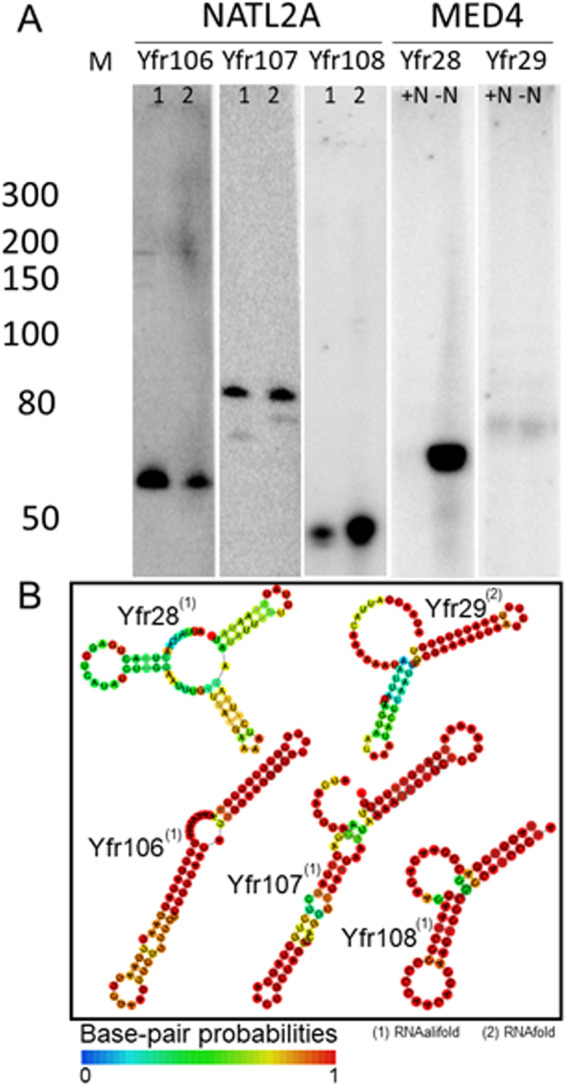
Experimental verification of environmentally relevant sRNAs and structure predictions. Verification of *Prochlorococcus* NATL2A and MED4 sRNA candidates using 32P-radiolabeled specific probes. **a** In the well-analyzed strain MED4, two new sRNAs (Yfr28 and Yfr29) were found, the first of which is only expressed under nitrogen starvation (-N). Hybridizations with NTAL2A were performed in technical duplicates (1 and 2). The numbers given with the marker (M) are sizes in nt. **b** The consensus structures of HL ecotype homologs of Yfr28 and of LL ecotype homologs of Yfrs106–108 were predicted with RNAalifold [56]. The structure of the MED4-specific sRNA Yfr29 was computed with RNAfold [57].

**Fig. 4:**
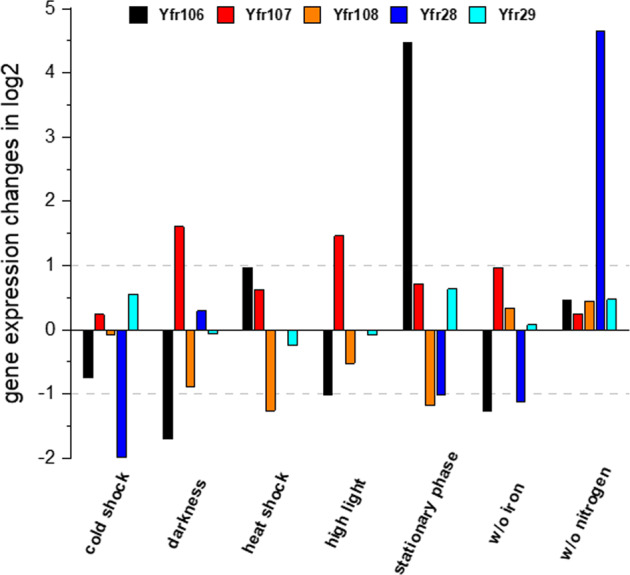
Expression responses of novel sRNAs to ambient perturbations. Cultures of *Prochlorococcus* strain NATL2A (Yfrs106–108) or MED4 (Yfr28 and Yfr29) were exposed to various stress conditions. Total RNA extracts were separated on PAA gels, transferred to Hybond N + membranes and hybridized with the respective probes. The fold changes (normalized against the internal standard 5 S rRNA) observed under different stresses in comparison with the control hybridization are given in log2 values. The horizontal dashed gray lines indicate the border of a ≥twofold change.

**Fig. 5:**
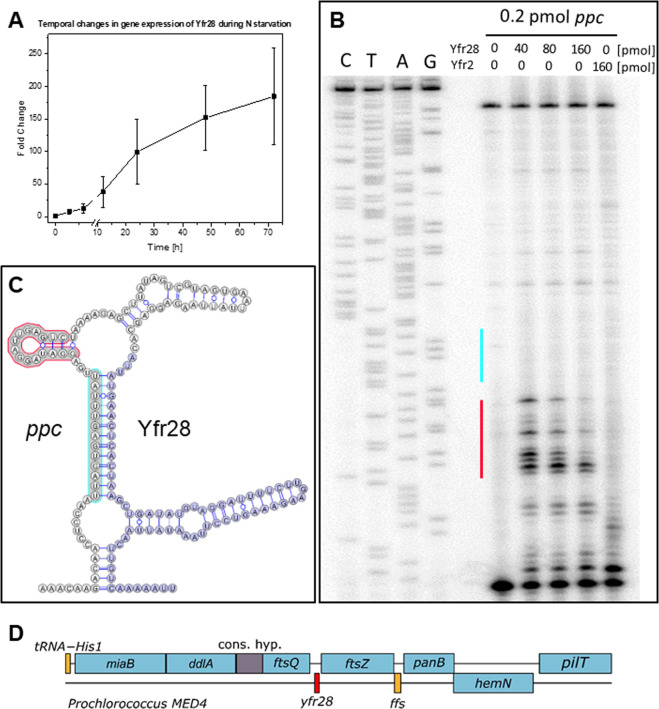
Temporal changes in Yfr28 expression during nitrogen starvation and target verification of *ppc*. **a** Yfr28 expression profile of *Prochlorococcus* MED4 samples after 0, 3, 6, 12, 24, 48, and 72 h of nitrogen starvation. The stress experiment was carried out in biological triplicates. Extracted RNA samples were separated on 7 M urea-10% PAA gels, transferred onto Hybond N+ membranes and hybridized with a Yfr28 probe. **b** Primer extension of 0.2 pmol of in vitro-synthesized 5′ region *ppc* mRNA in the absence of any synthesized sRNA or in the presence of varying amounts of Yfr28 or 160 pmol Yfr2. The vertical red line denotes the primer extension termination signals of *ppc* in the presence of Yfr28. The vertical cyan line indicates the interaction region predicted by intaRNA [58]. **c** Cofolded structures of *ppc* (white-circled nucleotides) and Yfr28 (royal blue-circled nucleotides). The red and cyan border lines of the nucleotides correspond to the vertical lines in (**b**). **d** Location of *yfr28* (red box) in the *Prochlorococcus* MED4 genome. Genes of known or unknown function are indicated in blue and gray boxes, respectively, and genes encoding small RNAs are indicated in orange boxes.

After the manual inspection of potential sRNAs, the most promising candidates were subjected to northern hybridization, and five of the tested candidates could be validated and showed typical sizes and structures (Fig. 3). In a recent study, we observed the *Prochlorococcus* HL and LL clade-specific occurrence of sRNAs [16]. Except for Yfr29, which was only present in MED4, all other newly detected sRNAs were *Prochlorococcus*- or even clade-specific, confirming our previous findings [16]. Because of their mode of action, sRNAs are often coregulated with the environmental conditions in which they play a role. With the exception of the MED4-specific sRNA Yfr29, all other sRNAs responded to tested environmental fluctuations such as variations in light intensity (Yfr107) and changes in temperature (Yfr28 and Yfr108), with the strongest responses being observed under nitrogen deprivation (Yfr28) and in the stationary phase (Yfr106) (Fig. 4). Interestingly, these were the same highly responsive conditions that we observed in our previous study [16]. Subsequently, we focused on the 72 nt-long sRNA Yfr28, which occurred in both subclades HLI (MED4 and MIT9515) and HLII (MIT9215, MIT9301, MIT9312, AS9601, and MIT0604). The *yfr28* gene is framed on the opposite strand by the *ftsQ* and *ftsZ* genes, encoding cell division proteins (Fig. 5d). The synteny is highly conserved (Fig. S1). Next, we predicted targets for Yfr28 using CopraRNA [37, 38] including all 7 *Prochlorococcus* strains with a Yfr28 homolog (Table S6). First, to gain a better understanding of the interaction of Yfr28 with its targets, we investigated the temporal expression kinetics of Yfr28 during nitrogen-limiting conditions (Fig. 5a). The expression of Yfr28 was induced sevenfold after 3 h of nitrogen depletion and continuously increased to almost 200-fold of the initial expression value within 72 h (Fig. 5a). This is the most strongly induced *Prochlorococcus*-related sRNA identified in response to nitrogen starvation to date. In a previous study, we observed a tenfold increase in response to nitrogen limitation for the highly conserved and highly abundant sRNA Yfr2 [39]. The expression profiles are quite similar for Yfr28 and Yfr2; however, the latter sRNA reached its maximum level after 48 h, whereas Yfr28 had not yet reached its peak expression level after 72 h. Second, we used primer extension to validate the interaction between Yfr28 and the phosphoenolpyruvate carboxylase mRNA (*ppc*, PMM1575), which was ranked as its third-best predicted target (Fig. 5b). We observed distinct termination signals of *ppc* that did not appear in the negative control reaction with Yfr2 and *ppc* (Fig. 5a). These results are in full agreement with the predicted Yfr28-*ppc* structure complex, indicating that the synthesis of *ppc* cDNA ceased when the interaction of the two RNAs started (Fig. 5c). The interaction site of Yfr28 is within the last 120 nt of the *ppc* reading frame (Table 1). While the majority of characterized bacterial sRNAs act on the ribosome binding site (i.e., the 5′ UTR immediately upstream of the start codon), there are several sRNAs that pair deeply within the coding sequence [40–42]. Alternatively, or in addition, the regulation of this mRNA species by Yfr28 might also affect the 3′ adjacent gene PMM1574, encoding a GNAT family acetyltransferase. Looking at other targets, we noticed the pronounced overlap between Yfr28 and the 5′ UTR of the neighboring *ftsZ* gene (Table 1, Figs. S1 and S2). This 5′ UTR derives from an alternative promoter and is perfectly complementary to the first 46 nt of Yfr28 (Fig. S2). Other sRNAs with a regulatory function on *ftsZ* have been described in *Escherichia coli* [43] and *Sinorhizobium meliloti* [43, 44]. In *Escherichia coli*, it was shown that the prophage-encoded sRNA DicF inhibits cell division via direct base pairing with *ftsZ* mRNA to repress translation and prevent new synthesis of the protein. Robledo et al. demonstrated that *Sinorhizobium meliloti* sRNA EcpR1 posttranscriptionally modulates the regulation of cell cycle genes under detrimental conditions [44]. Among the top targets of Yfr28 is another cell division-related gene, the *trans* target *minE*, which encodes an ATPase-activating protein in the MinCDE system (Table 1). Interestingly, *minD*, encoding a membrane-bound ATPase, was in the top 40 list of EcpR1 [44]. Collectively, our data suggest that Yfr28 might play an important regulatory role affecting the cell division genes *ftsZ* and *minE* as well as *ppc* during nitrogen limitation. In addition, Yfr28 might be involved in the regulation of the alternative sigma factor *sigD* (PMM0577), which is the top five predicted target of Yfr28. The *sigD* homolog in *Synechocystis* sp. PCC6803 is *sll2012*, which was shown to be beneficial during nitrogen starvation, most probably because it ensures active function of genes required for maximal protection against oxidative stress and for keeping photosynthesis active [45].

**Table 1:** List of the top 20 MED4 Yfr28 targets predicted by CopraRNA [37, 38].

Gene ID	Old gene ID	Gene name	Function	Functional category	*P* value	Energy	Target interaction region start end	
tx50_rs07025	PMM1309	*ftsZ*	cell division protein	Cellular processes, Cell division	2.15E + 04	−16.135	1	17
tx50_rs01100	PMM0213	*sbtA*	sodium-dependent bicarbonate transport family permease	Transport and binding proteins	4.26E + 08	−7.1387	755	763
tx50_rs08435	PMM1575	*ppc*	phosphoenolpyruvate carboxylase	Energy metabolism	2.52E + 09	−10.2167	2902	2914
tx50_rs01095	PMM0212	*sbtB*	P-II-superfamily regulator	Regulatory functions	7.58E + 09	−6.41062	7	14
tx50_rs03080	PMM0577	*sigD*	DNA-directed RNA polymerase sigma-70 factor	Transcription	0.000177539	−10.3455	51	60
tx50_rs03225	PMM0606	*lepB*	signal peptidase I	Cellular processes	0.000236172	−7.52996	637	647
tx50_rs08385	PMM1566	*crtD*	C-3′ 4′ desaturase	Hypothetical	0.000439155	−5.39939	1434	1443
tx50_rs00980	PMM0189	*mnmE*	tRNA uridine-5-carboxymethylaminomethyl (U34) synthesis GTPase	Regulatory functions	0.000521394	−7.01056	37	48
tx50_rs05200	PMM0977	*urtE*	multidrug ABC transporter permease	Transport and binding proteins	0.000769967	−10.613	832	842
tx50_rs08200	PMM1529	*prfA*, *sueB*	peptide chain release factor RF-1	Other categories	0.000733699	−7.94757	649	657
tx50_rs04445	PMM0830	*folP*	dihydropteroate synthase	Biosynthesis of cofactors, prosthetic groups, and carriers	0.001278001	−12.8874	824	842
tx50_rs00380	PMM0072	*sufC*	Fe-S cluster assembly ATPase	Transport and binding proteins	0.001447766	−5.32535	726	732
tx50_rs03220	PMM0605		DUF760 domain-containing protein	Hypothetical	0.001670637	−8.43848	7	17
tx50_rs02615	PMM0485			Hypothetical	0.001817716	−7.53348	75	90
tx50_rs05485	PMM1943			Hypothetical	0.002320793	−7.11648	2	10
tx50_rs01665	PMM0320	*minE*	cell division topological specificity factor	Cellular processes, Cell division	0.002271225	−7.31318	160	168
tx50_rs03625	PMM0682	*aroB*	3-dehydroquinate synthase	Amino acid biosynthesis	0.002229185	−5.14622	1078	1095
tx50_rs02290	PMM0425		cryptochrome/photolyase family protein	Hypothetical	0.002493323	−8.37338	692	701
tx50_rs02800	PMM0522	*pyrH*, *smbA*	uridylate kinase	Purines, pyrimidines, nucleosides, and nucleotides	0.003065727	−4.92973	538	548
tx50_rs08965	PMM1679	*ligB*	ATP-dependent DNA ligase	DNA replication, recombination, and repair	0.002958663	−8.08688	337	343

Potential interactions were predicted for the entire CDS region, including 50 nt upstream and downstream. The numbers for the target interaction regions refer to the nt positions of the respective genes (with 1 corresponding to the nt at position −50 with regard to the CDS and positions 51–53 corresponding to the start codon). The complete results are given in Table S6.

**Fig. 6:**
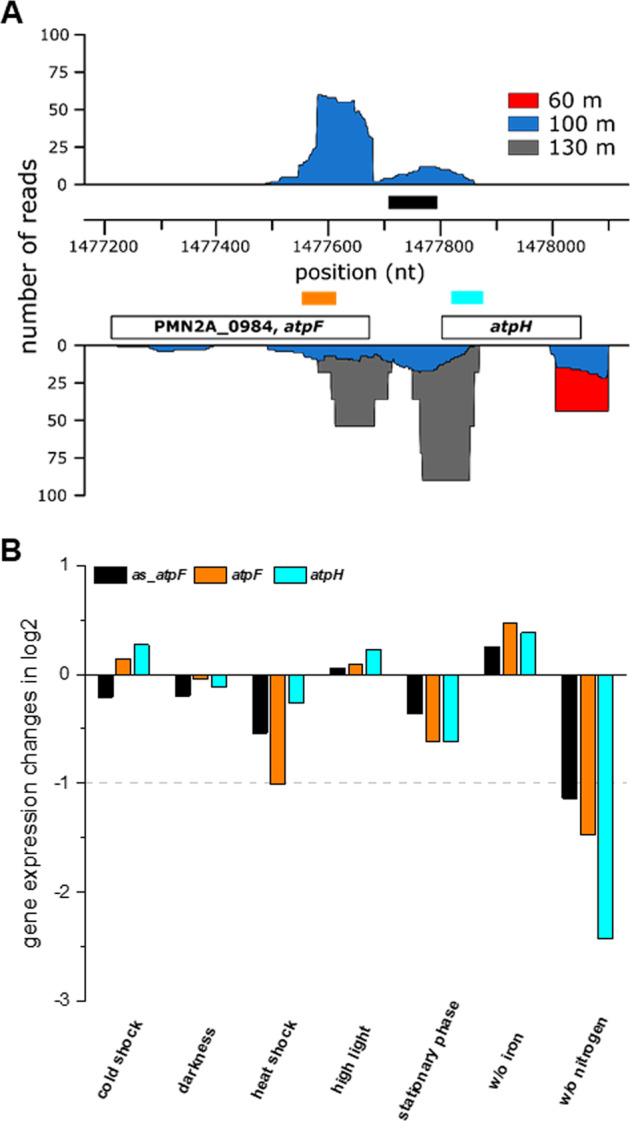
The gene expression of *atpF* is positively affected by a *cis*-encoded asRNA. **a** Coverage plots of NATL2A mapped read regions in the 60 m (red), 100 m (blue) and 130 m (gray) metatranscriptome samples. The white boxes correspond to the positions of the *atpF* and *atpH* genes (encoding the b’ and delta subunit, respectively, of the ATP synthase complex). The black, orange and cyan boxes denote the positions of the probes used for the northern hybridization of *as_atpF*, *atpF*, and *atpH*. **b** *Prochlorococcus* NATL2A cultures were exposed to various stress conditions, and total RNA extracts were separated on PAA gels, transferred to Hybond N + membranes and hybridized with the respective probes. The fold changes (normalized against the internal standard 5 S rRNA) observed under the different stresses in comparison with the control hybridization are given in log2 values. The horizontal dashed gray line indicates the border of a ≥two fold change. The northern blots that served as basis for this analysis are shown in Fig. S3.

One of the NATL2A sRNA candidates turned out to be an asRNA and is located opposite the IGR of *atpF* and *atpH* (encoding the b’ and delta subunits, respectively, of the ATP synthase complex), extending to the 5′ region of the *atpF* gene and the last 60 nt of the *atpH* gene (Fig. 6a). The 370 nt-long asRNA that we named *as*_*atpF* was most abundant in the 100 m sample (Fig. 6a), corresponding to the depth with the most NATL2A reads (Fig. 2). However, in northern hybridizations with total RNA from NATL2A cultures we also observed a ~70 nt long fragment (Fig. S3A) as well as longer, less distinct species of *as*_*atpF* (Fig. S3B). We further explored the functional mode of action of *as*_*atpF* by monitoring the differential expression of *as*_*atpF, atpF*, and *atpH* in NATL2A laboratory cultures during various stress conditions (Fig. 6b). The majority of tested stresses led to repression of the transcript levels of all three RNAs, and only modest induction of all three RNAs was observed upon HL treatment and iron starvation, suggesting that *as*_*atpF* stabilizes the mRNA of *atpF* and possibly also the mRNA of *atpH* (Fig. 6b). Furthermore, we noticed in some conditions the appearance of a short *atpF* mRNA fragment that is in the range of the coding sequence (462 nt) or slightly shorter, when ATP synthase might need to be stalled (especially darkness, stationary phase and cold shock, Figure S3B). This organization resembles the arrangement described for the *E. coli* asRNA GadY. GadY is transcribed from a promoter in the intergenic spacer between the genes *gadW* and *gadX* in antisense orientation to the 3′ end of *gadX*, which is an activator of the glutamate-dependent acid resistance system [46]. Upon binding of GadY, cleavage of the *gadXW* dicistronic mRNA is triggered [47], resulting in a more stable monocistronic *gadX* mRNA [48]. In MED4, similar transcriptional stress responses for *atpF* and *atpH* have been observed under darkness and HL exposure [29] and under nitrogen- [49] or iron-limiting conditions [50].

## Conclusions and possible implications

Bacterial ncRNAs are at the heart of regulatory pathways that allow bacteria to acclimate to changes in the environment, to adjust their metabolism, to regulate the expression of virulence genes and to control many other functions [51–53]. Here, we present a workflow for the identification of ncRNAs that appears to be particularly applicable to bacteria of high ecological relevance that are not amenable to direct manipulation. Starting with a metatranscriptomic dataset, we focused on the important primary producer *Prochlorococcus* and identified several ncRNAs that are likely relevant. The sRNA Yfr28 plays a pivotal role in the coordination of primary metabolism and cell division, as indicated by its very high low-nitrogen-induced expression and identified targets, which include mRNAs encoding the cell division proteins FtsZ and MinE, phosphoenolpyruvate carboxylase, the carbon uptake proteins SbtA and B and a sigma factor. The likely effect of controlling cell division and carbon metabolism under conditions of low-nitrogen supply is physiologically reasonable. Another presented example is the asRNA of *atpF*, which intriguingly showed, with the exception of cold shock, the same expression responses as the mRNA to typical environmental stress conditions. The presented workflow is of particular interest for environmentally relevant microorganisms, for which experimental manipulation ability might be limiting, while abundant sequence information may be available. All scripts utilized in this workflow are freely available.

## Supplementary information

Supplementary Table, Figure and File legends

Figure S1

Figure S2

Figure S3

Table S1

Table S2

Table S3

Table S4

Table S5

Table S6

Table S7

File S1

File S2
