# Supplementary material for: A framework for the computational prediction and analysis of non-coding RNAs in microbial environmental populations and their experimental validation
Source: ISME J. 2020 Apr 28;14(8):1955–65. doi: 10.1038/s41396-020-0658-7 (PMC7368042; doi:10.1038/s41396-020-0658-7)
Supplement: Supplementary file 1 — Supplementary Table, Figure and File legends [file 41396_2020_658_MOESM1_ESM.docx]

- 1. **Supplementary Tables, Figures and Files**

Table S1. List of oligonucleotides used in this study.

Table S2. List of computationally predicted sRNAs in NATL2A using RNAz [25] and of RFAM homologs [35, 36]. The results are sorted by the z-score computed with RNAz. The “prob-RNA” column lists the P-value for the predicted RNA class probability. Alignments with P>0.5 are classified as functional RNAs.

Table S3. List of computationally predicted sRNAs in MED4 using RNAz [25] and RFAM homologs [35, 36]. The results are sorted by the z-score computed with RNAz. The “prob-RNA” column lists the P-value for the predicted RNA class probability. Alignments with P>0.5 are classified as functional RNAs.

Table S4. Distribution of sRNA candidates in *Prochlorococcus* clades and other bacteria. The sRNA_IDs match those in Tables S2 and S3.

Table S5. Detection of known *Prochlorococcus* sRNAs in the 60 m, 100 m and 130 m samples using BLASTn. A BLAST search (with default settings) against all previously identified *Prochlorococcus* sRNA sequences was performed using the *Prochlorococcus*-associated non-CDS contigs (for more details, see the “Materials and Methods” section) from the three different depths (60 m, 100 m and 130 m) as query. Only BLAST hits with a coverage of at least 60% were considered as true positives.

Table S6. Computational target prediction for Yfr28 using CopraRNA [38, 39]. The entire CDS and 50 nt up- and downstream regions were searched for potential interactions. The analysis included *Prochlorococcus* strains MED4, MIT9515, MIT9215, MIT9301, MIT9312, AS9601 and MIT0604.

Table S7. Computational target prediction for Yfr106-108 using CopraRNA [38, 39]. The search region was set to 50 nt upstream and 250 nt downstream of the first nucleotide of the start codon. The analysis included *Prochlorococcus* strains NATL2A, NATL1A and MIT0801.

Figure S1. Synteny plot of the *yfr28* locus. The gene arrangement 3 kb up- and downstream is shown.

Figure S2. Overview of the read distribution in the *yfr28* and *ftsz* 5’ regions based on the mapped Solexa reads of primary cDNA reads. The blue boxes correspond to the gene position of *yfr28* and the position of the front part of the *ftsZ* gene. Sequence information for both strands is given, and -10 elements are indicated with yellow boxes. Transcriptional start sites are indicated in red letters, and an alternative start site for *ftsZ* is indicated in orange. The position of the transcriptional start site within the coverage plots is indicated by the dashed arrows. The initiator codon is shown as ATG. The putative interaction region of Yfr28 and *ftsZ* is indicated by the cyan line. Data were replotted from [16].

Figure S3. Northern blot hybridization results of *as_atpF*, *atpF* and *atpH* serving as basis for the analyses presented in Figure 6. A) Samples of various stresses (D – darkness, Fe – iron starvation, HL – high light stress, HS – heat shock, CS – cold shock, N – nitrogen starvation, C – control, Stat - stationary phase) were separated on PAA gels, transferred to Hybond N+ membranes and probed against *as_atpF* or 5S rRNA. B) Samples of the same stress conditions were separated on agarose gels, transferred to Hybond N+ membranes and probed against *as_atpF*, *atpF*, *atpH* or 5S rRNA. Note that in panel B the same membrane was used consecutively for all four hybridizations. C) Repetition of the nitrogen starvation experiment (biological replicate). D) Quantification of signals as in Figure 6B with the additional quantification of the separate short, possibly monocistronic signal for *atpF* (“*atpF_short*”). The log2 fold changes are given for each stress condition compared to the control and after normalization against the 5S rRNA level as internal standard. The areas used for quantification are indicated by the boxes outlined in red (for the “*atpF_short*” signal) or blue (all other conditions).

File S1. grp-file of the read distribution mapped to *Prochlorococcus* NATL2A for the 60 m library (first track), the 100 m library (second track) and the 130 m library (third track) on the forward strand. Data can be imported into the Artemis viewer.

File S2. grp-file of the read distribution mapped to *Prochlorococcus* NATL2A for the 60 m library (first track), the 100 m library (second track) and the 130 m library (third track) on the reverse strand. Data can be imported into the Artemis viewer.
