# Supplementary figures and images for: A framework for the computational prediction and analysis of non-coding RNAs in microbial environmental populations and their experimental validation
Source: ISME J. 2020 Apr 28;14(8):1955–65. doi: 10.1038/s41396-020-0658-7 (PMC7368042; doi:10.1038/s41396-020-0658-7)

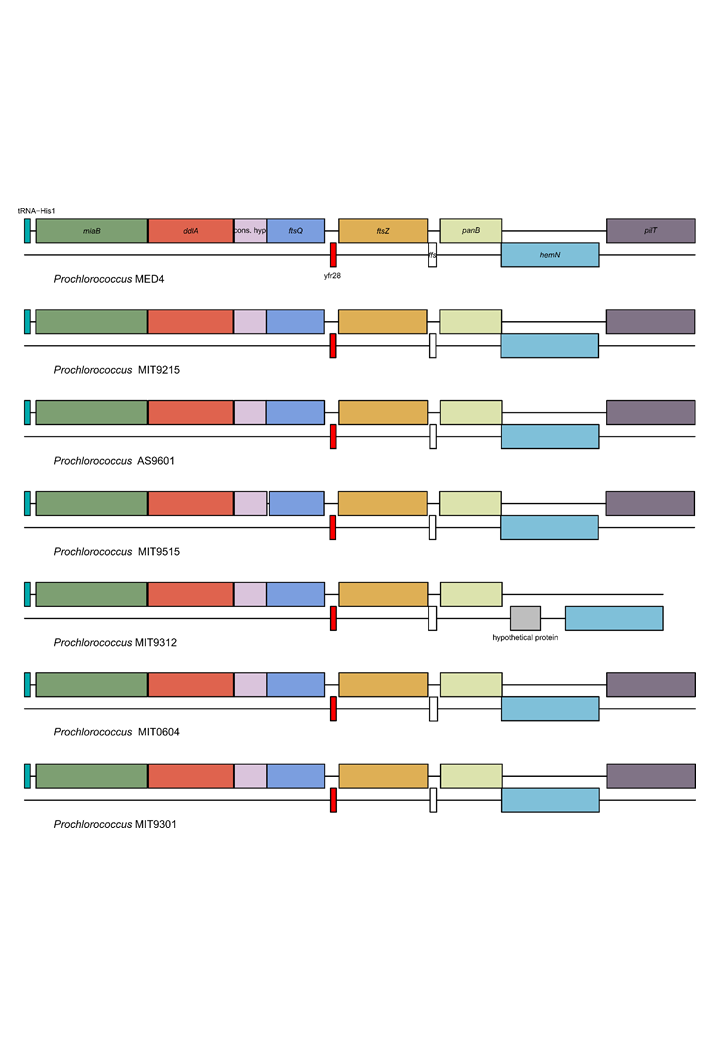

Supplement: Supplementary file 2 — Figure S1 [file 41396_2020_658_MOESM2_ESM.tif]

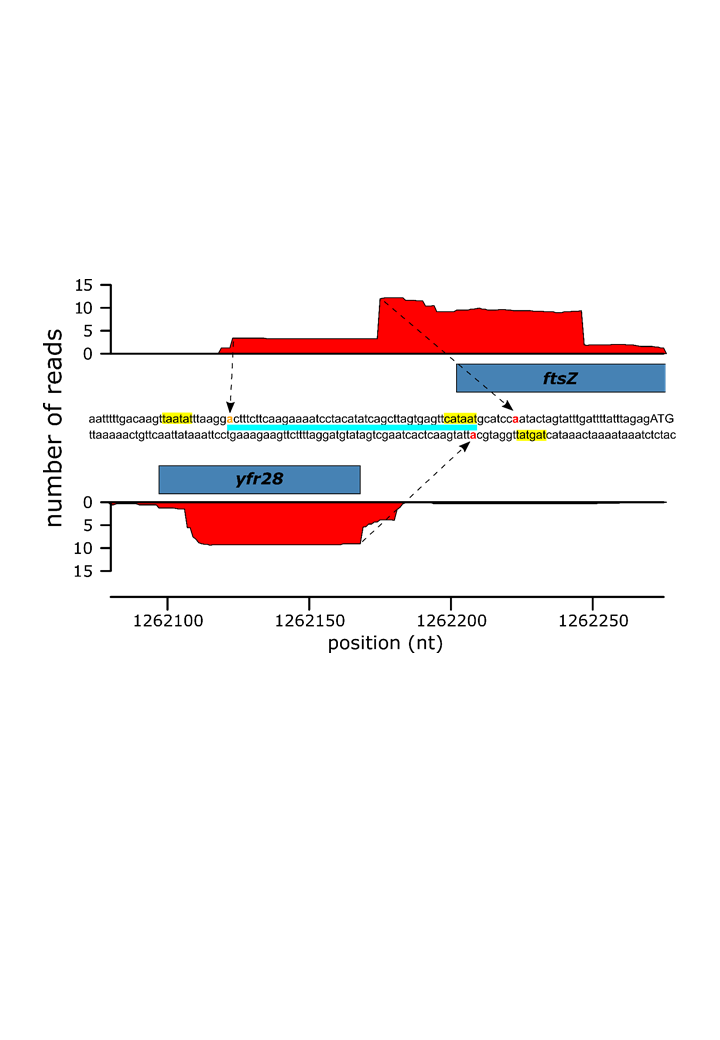

Supplement: Supplementary file 3 — Figure S2 [file 41396_2020_658_MOESM3_ESM.tif]

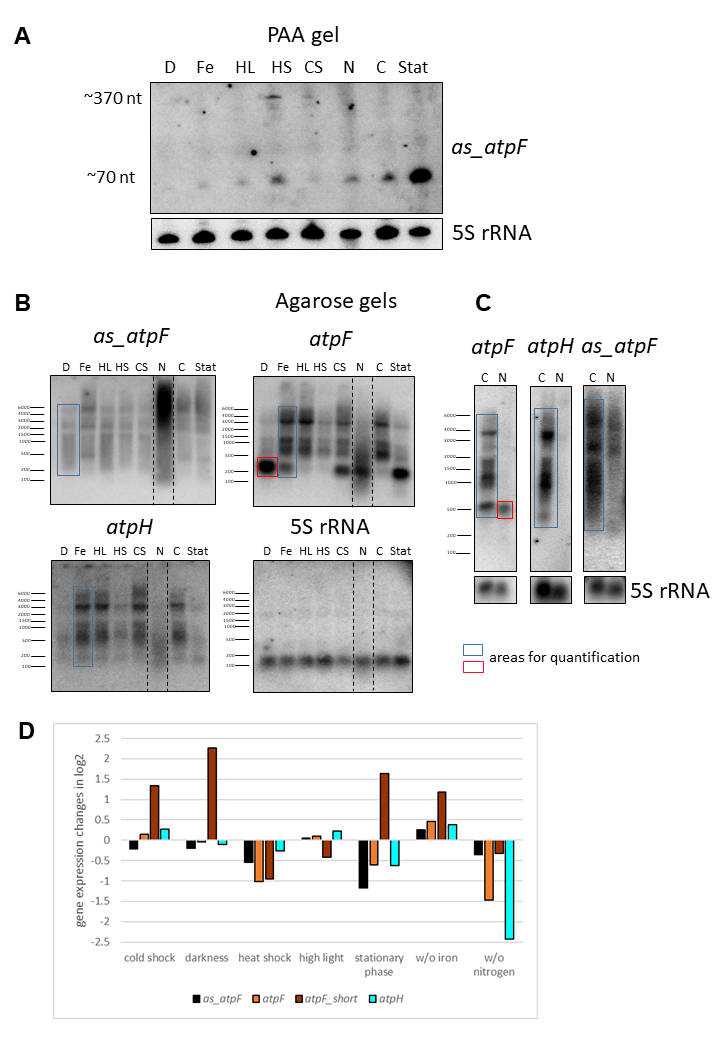

Supplement: Supplementary file 4 — Figure S3 [file 41396_2020_658_MOESM4_ESM.tif]
